# Curcumin Containing PEGylated Solid Lipid Nanoparticles for Systemic Administration: A Preliminary Study

**DOI:** 10.3390/molecules25132991

**Published:** 2020-06-30

**Authors:** Debora Santonocito, Maria Grazia Sarpietro, Claudia Carbone, Annamaria Panico, Agata Campisi, Edy Angela Siciliano, Giovanni Sposito, Francesco Castelli, Carmelo Puglia

**Affiliations:** Department of Drug Sciences, University of Catania, Viale Andrea Doria n°6, 95125 Catania, Italy; debora.santonocito@outlook.it (D.S.); mg.sarpietro@unict.it (M.G.S.); carbone@unict.it (C.C.); panico@unict.it (A.P.); agcampisi@gmail.com (A.C.); edysiciliano@hotmail.it (E.A.S.); giovanni.sposito@hotmail.it (G.S.); fcastelli@unict.it (F.C.)

**Keywords:** curcumin, PEGylation, stealth system, SLN, Turbiscan, TEM, ORAC, cryoprotector

## Abstract

Curcumin (CUR) has a wide range of pharmacological properties, including anti-inflammatory and antioxidant activities, and it can be considered a good candidate for the potential treatment of central nervous system (CNS) pathologies, although its use in clinical practice is compromised due to its high lipophilicity. Solid lipid nanoparticles (SLNs) are well-known nanocarriers representing a consolidated approach for the delivery of lipophilic compounds, but their systemic use is limited due their short half-life. The formulation of stealth SLNs (pSLNs) could be a valid strategy to overcome this limit. Curcumin-loaded-pSLNs were prepared by the solvent evaporation method. Formulation was characterized for their mean size, zeta potential, size distribution, and morphology. Drug antioxidant activity was evaluated by Oxygen Radical Absorbance Capacity (ORAC) assay. Finally, the obtained formulations were analyzed in terms of long-term stability. Curcumin-loaded-pSLNs showed good technological parameters with a mean particle size below 200 nm, as confirmed by TEM images, and a zeta potential value around −30 mV, predicting good long-term stability. Differential Scanning Calorimetry (DSC) analysis confirmed that PEG micelles interacted with the SLN surface; this suggests the location of the PEG on the pSLN surface. Therefore, these preliminary studies suggest that the produced formulation could be regarded as a promising carrier for the systemic administration.

## 1. Introduction

Curcumin (diferuloylmethane; CUR) is an active compound, extracted from rhizomes by *Curcuma Longa*, which has shown excellent therapeutic properties for the treatment of various diseases [1,2,3,4,5]. CUR is an excellent antioxidant that is able to prevent or slow down damage to cells caused by free radicals, as well as reactive oxygen species (ROS) and reactive nitrogen species (RNS) [6]. These free radicals are physiologically generated by cells as a “waste product” of the activity of the mitochondrial electron transport chain. In particular conditions, such as chronic inflammation, cancer, and neurological disorders, their accumulation causes the so-called “oxidative stress”, which is an imbalance between the excessive production of oxidizing factors (such as free radicals) and the decrease in antioxidant defenses. The damage caused by free radicals mainly affects DNA, genetic heritage, and mitochondria; moreover, the ROS activates NF-κB, which sets in motion the transcription of a cascade of pro-inflammatory cytokines (TNF-α), IL-1β, IL-2, and IL-6, chemokines—IL-8, and adhesion molecules, such as ICAM-1, which are central mediators in the inflammatory response [7,8,9].

Due to its antioxidant (300 times higher than those of vitamin E) [10,11] and anti-inflammatory properties [12,13], CUR plays a significant beneficial and regulatory pleiotropic role in various pathological conditions such as cancer, cardiovascular diseases, Alzheimer’s disease, inflammatory disorders, and neurological disorders. CUR has also been proved to exert other activities of extreme interest, including anticoagulant, antithrombotic, antihypertensive, antidiabetogenic, hypocholesterolemic, antiviral, and hepatoprotective [14]. It could also be effective in the treatment of malaria and in the prevention of cervical cancer. Moreover, it can interfere with the replication of HIV [15], and it acts as a radical scavenger, inhibiting lipid peroxidation [16]. Although it has numerous potential therapeutic properties, CUR shows many disadvantages when administered in vivo such as poor bioavailability, absorption, and a very fast metabolism and elimination [17,18].

Therefore, innovative strategies are required in order to overcome the unfavorable features of this interesting natural active compound and to allow its potential application in a specific therapeutic field. The advent of nanotechnology has made important innovations in the field of biological research and clinical practice for systemic use. In particular, nanocarriers can be exploited to modify the release of drugs (allowing therapeutic agents to be selectively directed to a specific organ) and to improve the stability of the drug over time, avoiding its rapid degradation [19].

Solid lipid nanoparticles (SLNs) represent an alternative colloidal carrier system to polymeric nanoparticles, liposomes, and nanoemulsions [20]. SLNs are able to solubilize hydrophilic and lipophilic molecules in a physiologic environment, controlling their release and protecting them from degradation [21,22]. Compared to other systems, SLNs have many benefits such as ease of preparation, low cost, high-scale production, good physical stability, chemical versatility, good biocompatibility, and biodegradability of lipids [22]. Unfortunately, the systemic use of these colloidal carriers is limited by the presence of the Mononuclear Phagocytic System (MPS) and the Reticulo Endothelial System (RES) [23,24]. These endogenous systems recognize the nanocarriers as extraneous and carry them in the liver, spleen, lungs, and bone marrow (opsonization) [25,26,27], which is responsible for their short half-life (3–5 min) after intravenous administration. This effect can be exploited to target chemotherapy drugs to localize MPS tumors [21]. The nanoparticles locatization into other organs is not a simple task. As reported in the literature, PEGylation is a well-known strategy to produce SLN surface “stealth” (pSLN), thus allowing the nanocarriers to avoid early opsonization, prolonging their circulation time in the blood [28,29,30,31]. Therefore, the PEGylation of nanocarriers increases the intracellular bioavailability of the drug and leads to a better therapeutic efficacy [32].

The aim of this preliminary study is to obtain curcumin-loaded PEGylated SLN (CUR-pSLNs), for the potential treatment of CNS pathologies involving a deficit of antioxidant defenses (i.e., Alzheimer’s disease, neurological disorders, macular degeneration) by systemic administration. SLNs were PEGylated using DSPE-PEG_2000_ (1,2-distearoyl-sn-glycero-3-phosphoethanolamine-*N*-[amino (polyethylene glycol)-2000] ammonium salt), an FDA-approved polymer [33]. CUR-pSLNs were characterized in terms of mean size, zeta potential, size distribution, morphology, and long-term stability. Furthermore, DSC studies were performed in order to assess the degree of the PEGylation on the pSLN surface. Finally, the ORAC test was carried out to evaluate the effect of drug encapsulation on CUR antioxidant activity.

## 2. Results and Discussion

### 2.1. In Vitro Study

CUR-pSLNs were prepared using Compritol^®^ 888 ATO (glycerol behenate) as lipid matrix. This lipid was selected after a screening carried out by an in vitro assay able to determine the potential cytotoxic effects of the excipients on the neuronal cell line Dental Pulp Stem Cell (DPSC). We decided to conduct our research on a neuronal cell line, since the next aim will be to evaluate the effect of CUR in the treatment of important CNS pathologies involving a deficit of antioxidant defense (i.e., Alzheimer’s disease, neurological disorders). This lipid screening was done by preparing the blank SLNs using different lipid matrices such as stearic acid, Compritol 888 ATO, and Precirol 5 ATO; the lipid concentrations were chosen based on the amount we used to prepare pSLNs. Subsequently, we investigated the effect of these nanocarriers on the cell viability of adult primary stem cells [34,35]. We chose Dental Pulp Stem Cells (DPSCs) transdifferentiated in Neural Stem Cells (NSCs) because they possess protective effects against models of neurodegenerative diseases, including Alzheimer’s disease [36].

**Figure 1:**
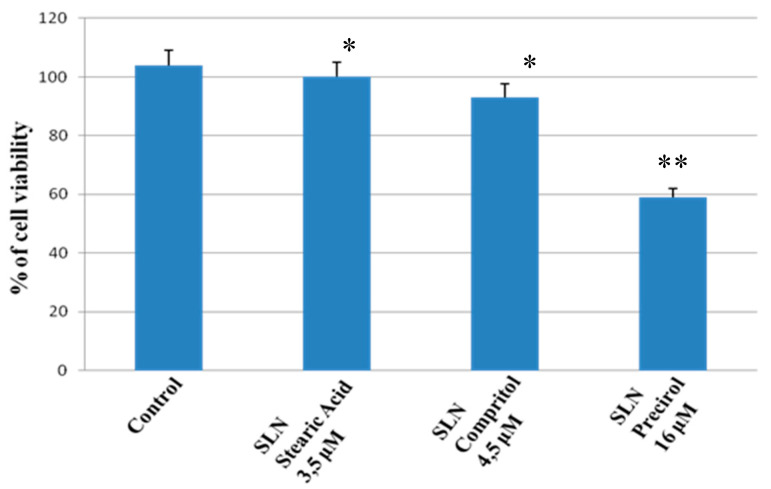
Percentage of viability of primary human Dental Pulp Stem Cell (DPSC) cell cultures exposed to stearic acid (3.5 µM), Compritol (4.5 µM) 888 ATO, and Precirol (16 µM) 5 ATO for 24 h. Results are expressed as the mean ± S.D. of the values of five separated experiments performed in triplicate. ** indicate *p* < 0.05 significant differences vs. control, and * represents *p* > 0.05 vs. control (untreated cells).

As reported in Figure 1, no significant changes in the percentage of cell viability in the cell cultures exposed to stearic acid and Compritol 888 ATO was observed with respect to the control, while Precirol 5 ATO induced a cytotoxic effect in DPSC cell cultures. Notwithstanding the similar effect of Compritol 888 ATO and stearic acid, we decided to formulate CUR-pSLNs using Compritol 888 ATO as a solid lipid due to the interesting results of Dynamic Light Scattering (DLS) characterization of blank SLNs.

### 2.2. SLNs Characterization

As reported in Table 1, DLS data showed good technological parameters with a mean diameter ranging from 130 to 180 nm, polydispersity index (PDI) values around 0.24–0.26, and zeta potential (ZP) values were in the range of −31 to −24 mV, predicting a good long-term stability. The slight difference in terms of mean particle size (10–20 nm) between SLNs and pSLNs was probably due to the PEG chains that fit into the surface of the nanoparticle, giving it an umbrella structure. The encapsulation efficiencies of CUR-SLNs and CUR-pSLNs were determined through a tangential ultrafiltration system, and they were approximately 82%.

**Figure 2:**
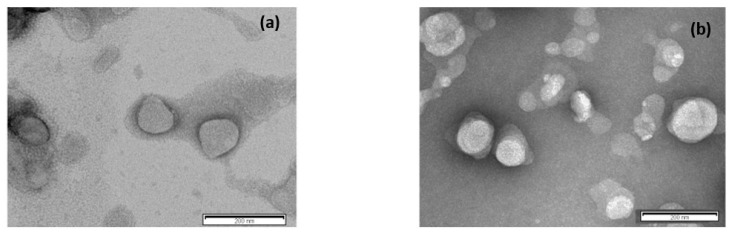
Transmission electron microscopy images of (**a**) blank pSLNs and (**b**) CUR-pSLNs. The scale bar represents 200 nm.

The morphology of blank SLNs and CUR-pSLNs were determined using TEM (Figure 2). TEM images showed that lipid nanoparticles had a spherical appearance having a particle size below 200 nm, as confirmed by DLS data.

### 2.3. Stability Studies

#### 2.3.1. Long-Term Stability

**Figure 3:**
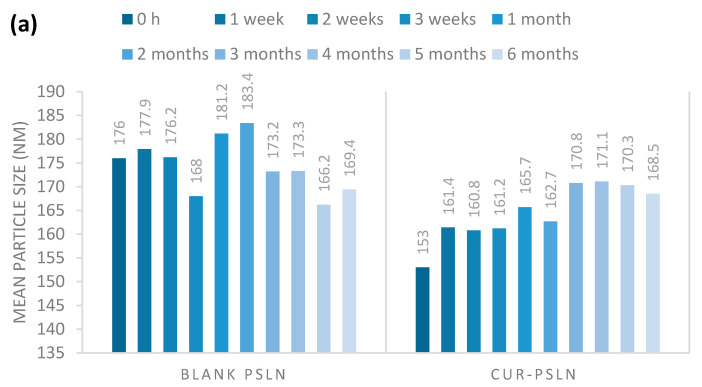

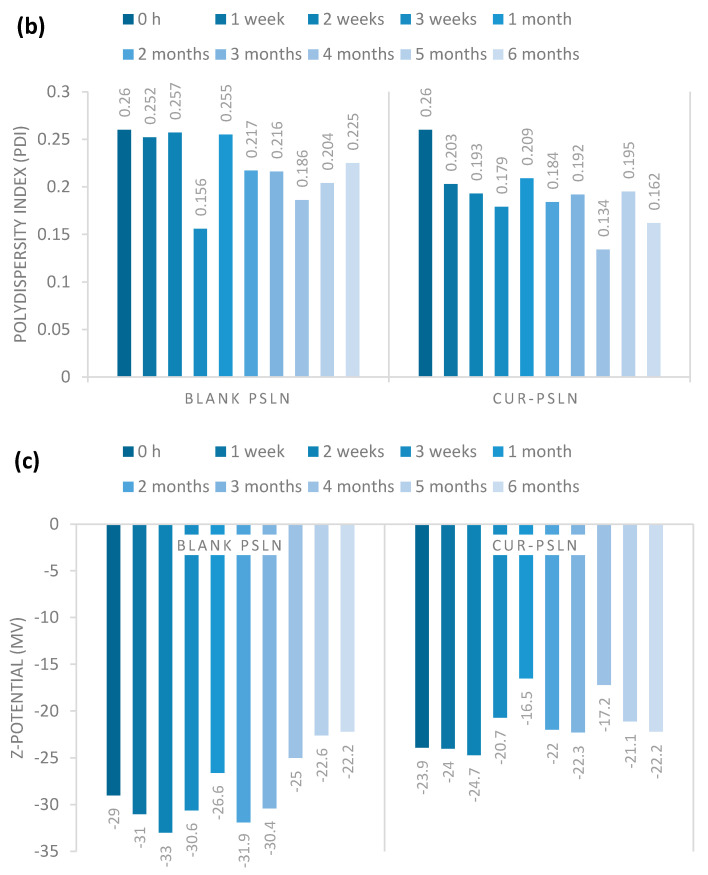
(**a**) Particle size, (**b**) polydispersity index (PDI), and (**c**) Z-potential of blank pSLNs and CUR-pSLNs during storage at room temperature for 6 months.

Stability of PEGylated nanoparticles was monitored for six months during storage at room temperature (Figure 3). In terms of stability, blank and CUR-pSLNs showed a good stability; in fact, no significant variation of mean size, homogeneity, and zeta potential values was observed in the DLS measurements after six months of storage at room temperature.

#### 2.3.2. Turbiscan Stability Studies

**Figure 4:**
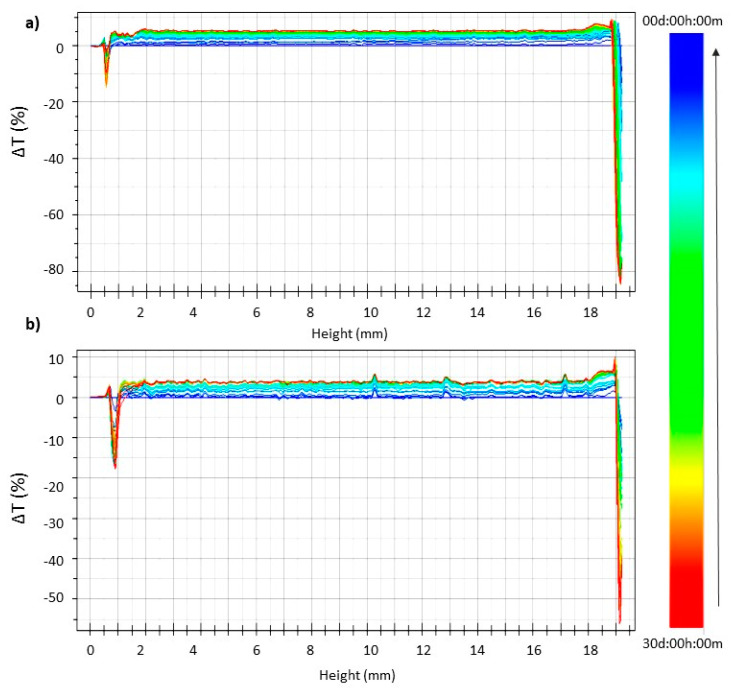
Turbiscan^®^ ΔT profiles of blank (**a**) and CUR (**b**) pSLNs stored for 30 days at 25 °C.

**Figure 5:**
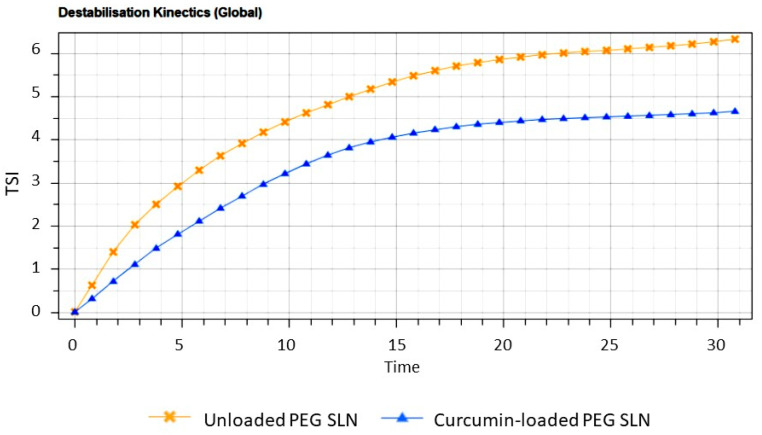
Destabilization kinetics in terms of Evolution of Turbiscan^®^ Stability Index (TSI) of blank and CUR-pSLNs stored for 30 days in TAGS at 25 °C.

Blank and CUR-pSLNs were stored at 25 °C for 30 days in Turbiscan^®^ station to evaluate the occurrence of physical instability phenomena: the occurrence of important variation in ΔT signal at the bottom or the top of the spectra indicates particles migration phenomena (clarification, sedimentation, or creaming), while variations in the middle are related to particle size changes. The transmission profile of blank and CUR-pSLNs are reported in Figure 4. The linearity of the transmission profiles (ΔT < 10%) clearly demonstrates the great physical stability during storage, without the occurrence of particles aggregation or migration at the top or at the bottom of the cuvette. The great physical stability could be related to the zeta potential values of the samples (around −30 mV). Destabilization kinetics reported as Turbiscan^®^ Stability Index (TSI) units (Figure 5) clearly showed that both samples were highly stable, since they did not show significant modification of the TSI value until 30 days of storage. However, based on the TSI global results, we could infer that CUR loading slightly increases the physical stability of the colloidal dispersion.

#### 2.3.3. Lyophilization Effect

A crucial point for the use of SLNs as colloidal drug carriers is their physical (aggregation/fusion of particles) and/or chemical (drug loss, microbiological contamination) stability due to their storage for long periods in aqueous medium [37,38,39]. In order to overcome this problem, it is highly desirable to have a freeze-dried SLNs formulation. A key role of the freeze-drying process is played by the cryoprotectant, which is able to immobilize the nanoparticles within its glass matrix, preventing their aggregation and protecting SLN against the mechanical stress of ice crystals [40].

**Figure 6:**
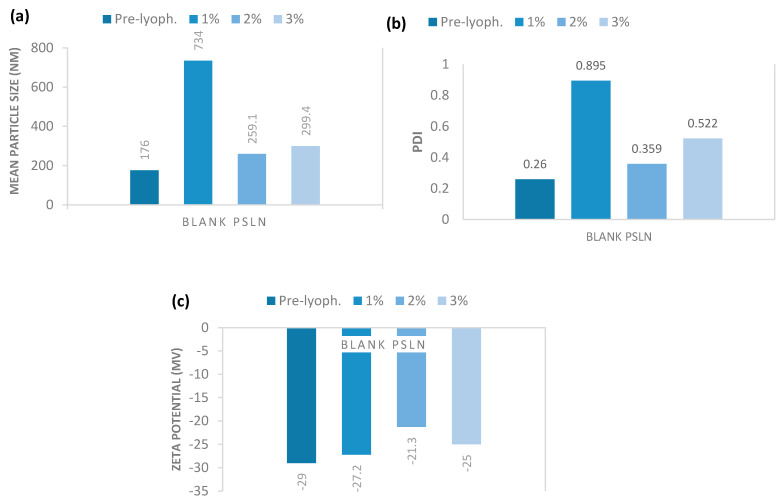
(**a**) Mean particle size (Z-Ave), (**b**) polydispersity index (PDI), and (**c**) zeta potential of blank pSLNs using increasing concentrations of glucose.

Therefore, in a pre-formulation study, we screened the ideal cryoprotectant between glucose, mannitol, and trehalose, following the evidences reported in literature, which identified these compounds as the most effective in protecting nanoparticles during the freeze-drying process [41,42,43]. The obtained results demonstrated that trehalose and mannitol in different concentrations (1–3% *w*/*w*) were not effective as cryoprotectants. In fact, all the samples dispersed after lyophilization, showed a significant increase in both mean particle size, ranging from 5119 to 8081 nm, and PDI values that were around 1.000. On the contrary, very promising results were obtained using glucose as cryoprotectant at the same tested concentrations. As reported in Figure 6, data demonstrated that 2% (*w*/*w*) of glucose is the best percentage to obtain a cryoprotectant effect for blank pSLNs during lyophilization. Similar results were observed for CUR-pSLNs (data not shown).

### 2.4. Differential Scanning Calorimetry (DSC)

**Figure 7:**
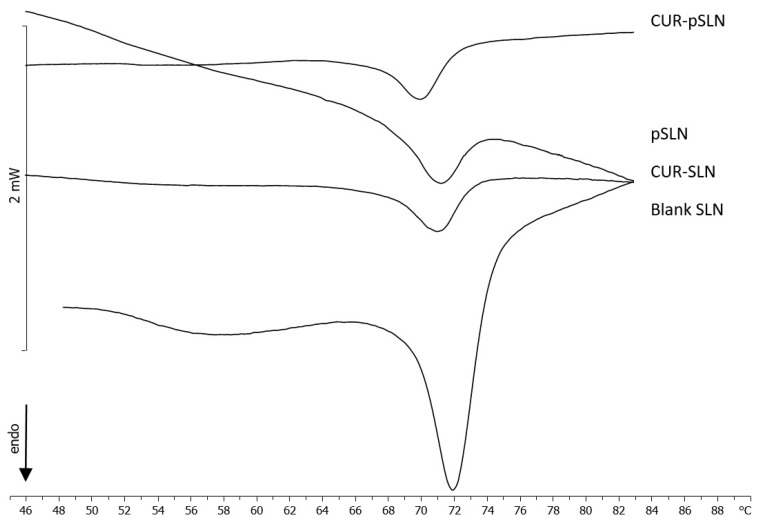
Calorimetric curves, in heating mode, of blank SLNs, CUR-SLNs, blank pSLNs, and CUR-pSLNs.

DSC studies were performed in order to assess the degree of the PEGylation on the pSLN surface. In Figure 7, the calorimetric curves of blank SLNs, CUR-SLNs, blank pSLNs, and CUR-pSLNs are reported. PEG exerted a remarkable effect on blank SLNs; in fact, the large shoulder almost vanished, and the sharp peak moved to lower temperature and decreased in intensity. In CUR-SLNs, the sharp peak appeared at temperature lower than that of blank SLNs, and the shoulder decreased. CUR-pSLNs showed a smaller shoulder and a main peak to a lower temperature than other SLNs, while the peak intensity was lower than blank SLNs. These results indicated that CUR and PEG showed a similar effect on SLNs; both lowered the fluidity of the SLN structure and decreased the cooperativity of the molecules in the system, as indicated by the lower temperature and the decreased intensity of the peak. It might suggest that both are localized among the SLN molecules.

In order to achieve information among the interaction between PEG (free compound or PEG micelles) and SLNs, kinetic experiments were run at different SLN/PEG molar ratios (1:0.1; 1:0.25; 1:0.5; 1:0.75; 1:1).

**Figure 8:**
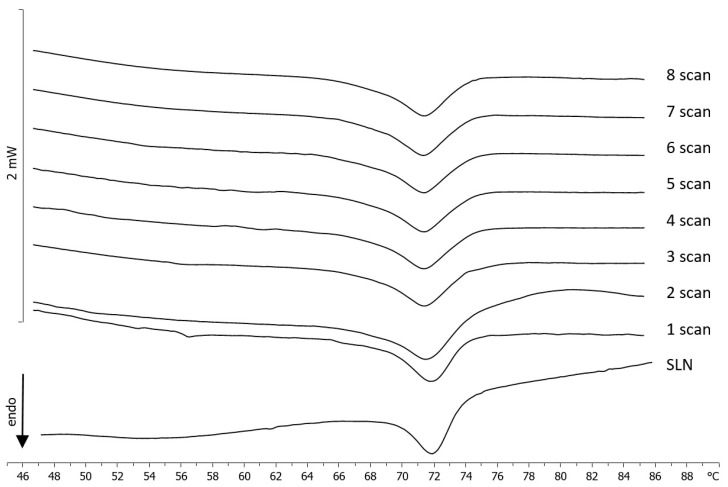
Calorimetric curves, in heating mode, of SLNs left in contact with PEG (1:0.5), at increasing incubation times.

PEG at different molar ratios was weighted in the bottom of the DSC calorimetric pan, SLNs were added, and the samples were submitted to calorimetric analysis. The calorimetric curves (referring to the molar ratio 1:0.5, the other molar ratios gave similar results) are shown in Figure 8. In these conditions, PEG did not exert a remarkable effect on SLNs, leaving the calorimetric curve almost unaffected; just a broadening of the main peak was visible. This result is clear evidence that free PEG does not interact with SLNs.

**Figure 9:**
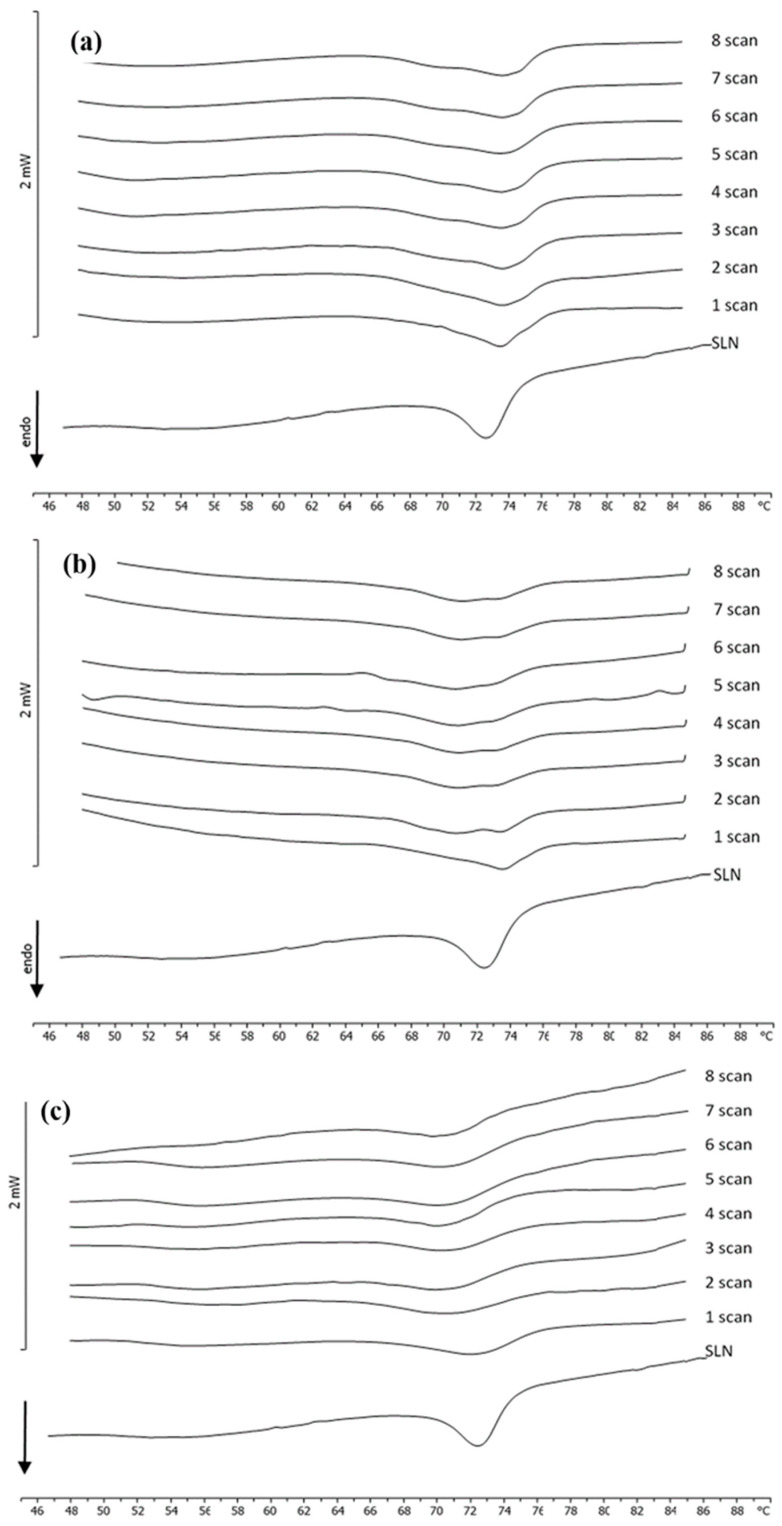
Calorimetric curves, in heating mode, of SLNs left in contact with PEG micelles (**a**) 1:0.1, (**b**) 1:0.5, and (**c**) 1:1 at increasing time of incubation.

Blank SLNs were put in the calorimetric pan, and PEG micelles were added at different molar ratios. The samples were immediately submitted to calorimetric analysis (Figure 9). It was clearly evident that PEG affects the SLNs’ calorimetric curve in different ways, depending on the molar ratio. In Figure 8, the calorimetric curve of blank SLNs are compared with the calorimetric curves of SLNs put in contact with PEG micelles at different incubation times (scan) (to save space, only the most representative calorimetric curves corresponding to 1:0.1, 1:0.5, and 1:1 molar ratios are shown). SLNs show a main peak at about 72.5 °C. At a 1:0.1 SLN/PEG molar ratio, the main peak is still present; however, a shoulder at about 69 °C is also visible. At a 1:0.25 molar ratio, the main peak decreases, and the shoulder increases. These results indicate that PEG interacts with the SLN and localizes on the outer layer of the SLN; in particular, the stearoyl moieties of the DSPE could be inserted on the SLN; whereas the PEG moiety could protrude on the SLN surface. The presence of two peaks at some amount of PEG micelles indicates that in the outer layer of the SLN, two different regions coexist: PEG-rich regions and PEG-poor regions. In particular, the signal at lower temperature is related to the PEG-rich regions, whereas the signal at higher temperature is related to the PEG-poor regions. On increasing the amount of PEG micelles, we observed that the peak at higher temperature decreased in favor of the peak at lower temperature and hence, in the SLN outer layer, the PEG poor regions decreased, whereas the PEG rich regions increased. At a 1:1 molar ratio, the presence of a single peak at the lower temperature suggests that PEG could be homogeneously present in the SLN outer layer; this could promote the CUR release in the case of CUR-SLNs.

### 2.5. In Vitro Antioxidant Activity

Oxygen Radical Absorbance Capacity (ORAC) assay has been widely accepted as a standard in vitro test to measure the antioxidant activity of natural active compounds [44,45]. In particular, this assay measures the loss of fluorescence over time due to peroxyl-radical formation by the breakdown of AAPH (2,2′-azobis-2-methyl-propanimidamide, dihydrochloride). Trolox [6-Hydroxy-2,5,7,8-tetramethylchroman-2-carboxylic acid], a water-soluble vitamin E analogue, is used as a positive control, inhibiting fluorescein decay in a dose-dependent manner. In our experiments, the difference between the “area under the fluorescence decay curve” (AUC) in the presence and in the absence of an antioxidant was translated into a Trolox standard calibration curve to express the antioxidant activity as Trolox equivalent for µM (TE/µM) of free CUR and CUR-pSLNs.

**Figure 10:**
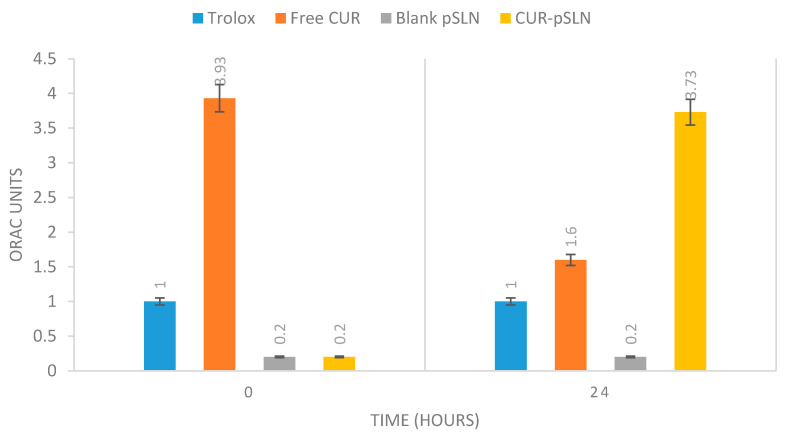
Oxygen Radical Absorbance Capacity (ORAC) activity of Trolox, curcumin, blank pSLNs and CUR-pSLNs. Trolox = 1 Units ORAC.

The results reported in Figure 10 pointed out the effect of encapsulation on CUR antioxidant activity. In particular, at 0 h, free CUR showed a remarkable antioxidant activity with respect to encapsulated CUR. After 24 h of monitoring, free CUR antioxidant activity decreased, while the activity of CUR encapsulated in pSLNs significantly increased. These results confirm the evidence reported in the literature regarding the key role of SLN encapsulation in preserving the antioxidant capacity of active antioxidant compounds for a longer time, with a mechanism probably related to the preservation of the compound stability [46].

The promising results of the present work stimulate future research directions to test in vivo the efficacy of CUR-pSLNs in the treatment of important CNS pathologies involving a deficit of antioxidant defenses.

## 3. Materials and Methods

### 3.1. Materials

Compritol 888 ATO (COMP, MW 414.7 g/mol), a mixture of mono-, di-, and triglycerides of behenic acid, and Precirol ATO 5 (Glyceryl palmitostearate) were obtained from Gattefossè (Milan, Italy); Lecinol S-10, hydrogenated lecithin, was obtained from Nikko Chemical (Milan, Italy), and Lutrol F68 (MW 8400 g/mol) were provided by BASF ChemTrade GmbH (Burgbernheim, Germany). DSPE-PEG_2000_ (1,2-distearoyl-sn-glycero-3-phosphoethanolamine-*N*-[amino (polyethyleneglycol)-2000] ammonium salt) was a gift from Lipoid GmbH (Ludwigshafen, German-y). Curcumin (CUR, MW 368.38 g/mol), Trolox (MW 250.29 g/mol), AAPH (2,2′-azobis(2-methylpropionamidine) dihydrochloride, MW 271.19 g/mol), Stearic Acid (284.48 g/mol), and all reagents were purchased from Sigma-Aldrich (St. Louis, MO, USA). Fluorescein disodium salt (MW 332.31 g/mol) was obtained from Acros Organics. Dulbecco’s Modified Eagles Medium (DMEM), heat-inactivated Fetal Bovine Serum (FBS, Gibco, Rockville, MD, USA), Penicillin/Streptomycin solution, 200 mM L-glutamine, collagenase, 0.05% trypsin-0.02% EDTA solution were from Invitrogen (ThermoScientific, Milan, Italy). Cytosine arabinoside, 1-(4,5-Dimethylthiazol-2-yl)-3,5-diphenylformazan (MTT), Dimethyl Sulfoxide (DMSO), and other chemicals were from Sigma-Aldrich (Milan, Italy).

### 3.2. Cell Culture

#### 3.2.1. Primary DPSC Cell Culture

The Dental Pulp Stem Cells (DSPCs) were isolated from human teeth. Freshly extracted teeth were immediately cracked open, and pulp tissue was collected, minced into small fragments of 1 mm, and then digested in 3 mg/mL collagenase type I for 1 h at 37 °C. The tissue pellet, after filtration by using a 70-μm filter, was resuspended in DMEM containing 15% (*v*/*v*) FBS, 2mM L-glutamine, 50 mg/mL penicillin (50 U/mL), and it was plated in 75 cm^2^ flasks at a final density of 2 × 10^6^ cells or placed at the final density of 0.5 × 10^4^ cells/well of a 96-multiwell plates. When the cultures were about 80% to 85% confluent, cells were trypsinized by using 00.05% trypsin-0.02% EDTA solution. After centrifugation, cells were resuspended in fresh basic complete media, plated at a 1:4 density ratio in 75 cm^2^ flasks, and incubated at 37 °C in humidified atmosphere containing CO_2_ (95–5%), and the medium was replaced every 2 or 3 days.

#### 3.2.2. Treatment of Cell Cultures

The DSPC cell culture was suspended in the specific complete culture medium and placed in 96-multiwell plates and placed for at 37 °C in a humidified atmosphere and CO_2_ (95–5%). In order to establish the optimal lipid matrix to formulate CUR-pSLNs, cell cultures were treated for 24 h.

#### 3.2.3. MTT Assay

Cell survival analysis was performed by MTT reduction assay, evaluating mitochondrial dehydrogenase activity. Cells were set up 0.5 × 10^4^ cells per well of a 96-multiwell, flat-bottomed, 200-mL microplate and maintained at 37 °C in a humidified CO_2_ (95-5%) air mixture. At the end of treatment time, 20 mL of 0.5% MTT in (pH 7.4) PBS were added to each microwell. After 1 h of incubation with the reagent, the supernatant was removed and replaced with 100 mL of DMSO. The optical density of each well was measured with a microplate spectrophotometer reader (Titertek Multiskan; Flow Laboratories, Helsinki, Finland) at λ = 570 nm. Results were normalized with DMSO control (0.05%) and expressed as a percentage of cell viability inhibition.

#### 3.2.4. Statistical Analysis

Data were statistically analyzed using One-Way analysis of variance (ANOVA) followed by a post hoc Holm–Sidak test to estimate significant differences among groups. Data were reported as mean ± SD of four separated experiments in duplicate, and differences between groups were considered to be significant at * *p* < 0.05.

### 3.3. SLNs Preparation

SLNs were formulated following a valid and highly reproducible method, using Compritol^®^ 888 ATO (glycerol behenate) as lipid phase and Lutrol F68^®^ (Poloxamer 188) as surfactant. Nanoparticles loaded with curcumin (CUR-SLNs) were prepared by the *solvent evaporation* method with slight modifications [47,48]. Firstly, CUR (99 mg), Compritol 888 ATO (60 mg), and injectable soy lecithin (60 mg) were solubilized and melted in ethanol (10 mL) at 70 °C. For the preparation of PEGylated SLNs loaded with curcumin (CUR-pSLNs), 15 mg of DSPE-PEG_2000_ was added to the lipid phase. The melted lipid phase was dispersed in the hot (70 °C) surfactant solution (Lutrol F68, 0.5% *w*/*v*) under stirring at 695 rpm. Then, the hot dispersion was cooled in an ice bath (2–3 °C) for 5 min. Finally, the organic solvent was removed by vacuum. Blank nanoparticles were prepared by the same procedure without adding CUR.

### 3.4. SLNs Characterization

The average size (Z-Ave) and polydispersity index (PDI) were determined by Dynamic Light Scattering (DLS). A Zeta Sizer Nano-ZS90 (Malvern Instrument Ltd., Worcs, England), equipped with a solid-state laser having a nominal power of 4.5 mW with a maximum output of 5 mW 670 nm, was employed. Analyses were performed using a 90° scattering angle at 20 ± 0.2 °C. Samples were prepared diluting 100 μL of SLN suspension with 900 μL of distilled water. Each value was measured at least in triplicate.

The zeta potential (ZP, ξ) was measured by Electrophoretic Light Scattering (ELS) using the Zeta Sizer Nano-ZS90 (Malvern Instrument Ltd., Worcs, England). The instrument carried out three sets of measures up to 100 to achieve an average value. Samples were suspended in distilled water, and the measurements were recorded at 25 °C. Each value was measured at least in triplicate.

Morphological and structural characteristics of CUR-pSLNs were investigated using TEM (JEOL JEM-101).

### 3.5. Determination of Drug Loading

The unentrapped CUR was removed by filtration using a Pellicon XL™ tangential ultrafiltration system (Millipore, Milan, Italy) equipped with a polyethersulfone Biomax 1000 membrane with a 1,000,000 molecular weight cut off (MWCO). An amount of the lyophilized nanoparticles suspension was solubilized in dichloromethane, and the CUR content was measured by UV spectrophotometry at 425 nm (Lambda 52, PerkinElmer, MA, USA). Calibration curves for the validated UV assays of CUR were performed on six solutions in the concentration range 10–100 μg/mL (r^2^ > 0.99). Each point represented the average of three measurements, and the error was calculated as standard deviation (±SD). The entrapment efficiency of CUR in the lipid matrix was calculated from Equation (1):

Drug recovery (%) = Mass of active in nanoparticles/Mass of active fed to the system × 100
(1)

Possible lipid interferences during UV determination of CUR were studied between the standard curves of free CUR and in the presence of lipids. The differences observed were within the experimental error, thus inferring that no lipid interference occurred.

### 3.6. Stability Tests

#### 3.6.1. Long-Term Stability

Technological parameters (particle sizes, PDI, and ZP values) of SLN samples were measured over time at intervals (zero hour, one week, two weeks, three weeks, one month, two months, three months, four months, five months and six months). During storage, samples were maintained at room temperature and protected from light exposure.

#### 3.6.2. Samples Stability by Turbiscan^®^ Aging Station (TAGS)

The physical stability of SLN suspensions was evaluated by a Turbiscan^®^ Aging Station (TAGS) (Formulaction, l’Union, France). First, 20 mL of each sample were placed in a cylindrical glass cell and positioned in the Turbiscan^®^ at 25 °C for 30 days. Turbiscan^®^ AGS has been previously reported as a reliable technology to evaluate the occurrence of instability phenomena related to particle aggregation and/or migration [49,50,51,52,53,54]. In our experiments, the stability of the samples was evaluated based on the variation of transmission (ΔT), which is shown in ordinate, while the height of the cell is reported in abscissa. The transmission (T) detector receives the light, which crosses the sample (at 180° from the incident beam), using a pulsed near-infrared light source (λ = 880 nm). The detection head scanned the entire height of the sample cell (65 mm longitude), acquiring T signals each 40 mm (1625 acquisitions in each scan). The Turbiscan^®^ makes scans at various pre-programmed times and overlays the profiles on one graph in order to show possible destabilization phenomena.

#### 3.6.3. Freeze Drying of SLNs

Glucose, mannitol, and trehalose were tested to select the cryoprotectant and the most suitable concentration for the formulation. The SLN dispersions were added with each cryoprotectant, at different concentrations, before freezing. Then, 2 mL of these dispersions were placed in 5 mL vials. Freezing is the first step of freeze-drying; then, samples were lyophilized for 24 h. In order to rehydrate the freeze-dried nanoparticles, the same volume of water lost during lyophilization was added. After reconstitution by manual shaking, nanoparticles size was measured by DLS. The preservation of nanoparticle mean size after freeze-drying is considered as a good indication of a successful freeze-drying cycle [55].

### 3.7. PEG Micelles Preparation

Micelles of DSPE-PEG_2000_ were prepared using the *thin-layer evaporation* method [56,57]. They were prepared by dissolving the polymer in an organic solvent mixture (chloroform/methanol 1:1 *v*/*v*). Aliquots of PEG suspensions (which corresponded to 1:0.1, 1:0.25, 1:0.5, 1:0.75, and 1:1 molar ratios with respect to the lipid of SLNs) were placed in glass vials. To obtain a film, the solvents were firstly evaporated under a nitrogen stream, and then, samples were lyophilized to obtain a solvent-free film. Subsequently, the DSPE-PEG_2000_ films were hydrated using distilled water. The samples were kept at 80 °C for 1 min and vortex for 1 min, for two times, and then they were finally kept at 80 °C for 60 min.

### 3.8. Differential Scanning Calorimetry (DSC)

DSC analysis was carried out using a Mettler Toledo STAR^e^ system (Switzerland) equipped with a DSC-822 calorimetric cell. A MettlerTA-STAR^e^ software (16.00 version) was used to acquire and analyze data. The DSC was calibrated using indium (≥99.95% purity). The reference pan was filled with 120 μL of distilled water. Each sample (120 μL) was loaded into a 160 μL aluminum crucible, hermetically sealed, and submitted to DSC analysis, under an atmosphere of dry nitrogen. DSC analysis was carried out using a heating scan from 5 to 85 °C (2 °C /min) and a cooling scan from 85 to 5 °C (4 °C/min) for at least three times [58].

The degree of PEGylation of the pSLNs was evaluated by DSC analysis using SLNs blank and loaded with CUR. First, 30 μL of each DSPE-PEG_2000_ micelles were co-incubated with SLNs and submitted to DSC analysis. The scanning program consisted of a heating scan from 4 to 85 °C, an isothermal segment at 85 °C for 1 h, and a cooling scan from 85 to 4 °C. The analysis was repeated eight times to allow hour by hour to monitor the thermodynamic changes taking place in the SLNs.

### 3.9. Oxygen Radical Absorbance Capacity (ORAC) Assay

The antioxidant activity of CUR, free or loaded into pSLNs, was assessed in vitro using the ORAC assay, according to the procedure previously reported [59,60]. Data were obtained using a VICTOR Wallac 1420 Multilabel Counters fluorimeter (Perkin Elmer, Boston, MA, USA) with a fluorescence filter (excitation 540 nm, emission 570 nm). Fluorescein (FL) solution (12 nM) was the fluorescence probe and the target molecule for free radical attack by the peroxyl radical generator AAPH (100 mM). The assay was carried out at pH 7.0 and at 37 °C, using Trolox (12.5 μM) as the control standard and phosphate buffer (pH 7.0) as the blank. CUR was solubilized in ethanol (12.5 µM), while CUR-pSLNs were diluted at the same concentration with phosphate buffer. After adding AAPH, the fluorescence was recorded for 24 h. All measurements were expressed in relation to the initial reading, analyzing all samples, one blank and one standard at the same time. Each measure was performed in triplicate. The ORAC value refers to the net protection area under the quenching curve of FL in the presence of an antioxidant. The results (ORAC values) were calculated and were expressed using Trolox equivalents (TE) for μM of sample (TE/μM) according to Equation (2):

*ORAC units* (*TE*/*μM*) = *K* (*S_sample_* − *S_blank_*)/(*S_trolox_* − *S_blank_*) (2)

where *K* is a sample dilution factor and *S* is the area under the fluorescence decay curve of the sample, Trolox, or blank calculated with Origin^®^7 (OriginLab Corporation, Northampton, MA, USA).

## 4. Conclusions

This preliminary study is finalized to obtain an innovative drug delivery system loaded with CUR (CUR-pSLNs), which is an important antioxidant that could be used to treat several CNS pathologies involving a deficit of antioxidant defenses, suitable for systemic administration. CUR-pSLNs were prepared by the solvent evaporation method obtaining spherical nanoparticles having a particle size suitable for parenteral administration (<200 nm). Nanoparticles showed a good stability for six months at room temperature, as confirmed by Turbiscan Technology. Furthermore, the obtained data showed that the freeze-drying of CUR-pSLNs under optimized conditions lead to a lyophilized sample with good reconstitution properties, thus protecting the drug. Finally, CUR-pSLNs showed greater antioxidant activity over time than free CUR, confirming the key role of encapsulation in preserving and therefore increasing the antioxidant activity of powerful active compounds.

In conclusion, our results suggest that pSLNs could be regarded as a promising delivery system for CUR systemic administration. Works are in progress to test in vivo CUR-pSLNs efficacy in the treatment of important CNS pathologies involving a deficit of antioxidant defenses.

## Figures and Tables

**Table 1:** The values of Z-Ave, polydispersity index (PDI), and Z-potential for blank solid lipid nanoparticles (SLNs), blank stealth SLNs (pSLNs), curcumin-loaded PEGylated SLN (CUR-SLNs), and CUR-pSLNs recorded at 25 °C. ZP: zeta potential.

Formulation	Z-Ave [nm ± SD]	PDI [–] ± SD	ZP [mV ± SD]
Blank SLNs	161.8 ± 0.1	0.24 ± 0.1	−31.4 ± 0.3
Blank pSLNs	176.0 ± 0.2	0.26 ± 0.1	−29.0 ± 0.1
CUR-SLNs	132.0 ± 0.1	0.25 ± 0.0	−24.3 ± 0.2
CUR-pSLNs	153.3 ± 0.2	0.26 ± 0.2	−23.9 ± 0.1
